# Opportunities and Challenges of the Human Microbiome in Ovarian Cancer

**DOI:** 10.3389/fonc.2020.00163

**Published:** 2020-02-18

**Authors:** Huiyan Cheng, Zhichao Wang, Lifeng Cui, Yan Wen, Xiuhua Chen, Fengyan Gong, Huanfa Yi

**Affiliations:** ^1^Department of Gynecology and Obstetrics, The First Hospital of Jilin University, Changchun, China; ^2^Department of Pediatric Surgery, The First Hospital of Jilin University, Changchun, China; ^3^Central Laboratory of the Eastern Division, The First Hospital of Jilin University, Changchun, China

**Keywords:** ovarian cancer, human microbiome, 16S rRNA sequencing, chemotherapy, immune response, diagnosis, treatment

## Abstract

Ovarian cancer is the most lethal malignancy among gynecological cancers worldwide. Most ovarian cancer patients are diagnosed at an advanced stage because of non-specific clinical symptoms. The human microbiome plays a crucial role in maintaining the normal physiological and pathological state of the body. With the development of technologies such as DNA and 16S rRNA sequencing, an increasing number of findings on the role of microbiome in cancers are being reported. Microbiome abnormalities are increasingly associated with diseases, including cancer development, and response to therapies. Some studies have shown the relationship between microbiome changes and ovarian cancer. However, the mechanisms underlying this relationship are not yet fully understood. Here, we summarize the key findings in this regard by focusing on estrogen metabolism and host recognition receptors in microorganisms and changes in the gut or pelvic microbiome in patients with ovarian cancer. We further discuss the potential of using the microbiome as a novel biomarker for cancers. We also highlight the possibility to use microorganisms as a treatment modality to enhance the immune system, activate anti-tumor response, mediate chemotherapy resistance, and ameliorate the adverse effects of the treatment.

## Introduction

Ovarian cancer is the most lethal gynecological malignancy, with more than 200,000 new cases and 150,000 deaths reported annually worldwide (1). According to Surveillance, Epidemiology, and End Results (SEER) database, the incidence rates of new ovarian cancer cases and mortality rates of patients with ovarian cancer decreased annually by 1.9 and 2.2%, respectively, from 2004 to 2013 in the USA (2). Nevertheless, most cases of ovarian cancer are diagnosed at advanced stages, are resistant to front-line therapies, and show frequent relapse; thus, the treatment remains highly unsatisfactory. One of the proposed high-risk factors of ovarian cancer, particularly epithelial ovarian cancer, is lesions of the fallopian tube (3). The fallopian tubes are often affected by the pelvic inflammatory disease (PID), and inflammation of the fallopian tube is a contributor to infertility, conferring a high risk of ovarian cancer (4). Using high-throughput sequencing of 16S rRNA genes, Zhou et al. (5) have demonstrated a significantly reduced biodiversity and richness of the microbiome of ovarian cancer tissues compared to those of normal fallopian tube tissues, suggesting a link between microbiome and ovarian cancer.

The human microbiome is a complex ecosystem housing a variety of microorganisms residing mainly in the epidermal and mucosal tissues of the human body (6). The term “human microbiome” can be defined as the total of all microorganisms and their genomes residing in the human body and how they interact with the environment (7). The composition of the microbiome in the oral cavity, gut, vagina, pelvis, and other epithelial barriers can be shaped by host genes, gender, the delivery style at birth, age, microbial colonization at birth, and environmental factors such as diet, lifestyle, exposure to antibiotics, and diseases and their treatments (8). In addition, the microbiome can help to maintain normal host physiology, develop and educate the immune system, metabolize complex substrates, and provide key protection against opportunistic pathogens (9). Thus, a balanced microbiome composition can help to maintain a normal state of human health, and disturbance of this balance can lead to the development of various diseases, including cancer. On similar lines, changes in the intestinal and vaginal microbiome have been proposed to play a role in gynecological cancers such as cervical, uterine, and ovarian cancers (10); however, the precise pathological roles remain unclear. In this review, we focus on gathering evidence for a relationship between the microbiome and ovarian cancer and discuss the potential role of the microbiome in mediating the treatment effect of ovarian cancer.

## Human Microbiome

The entire human genome comprises ~25,000 human genes, whereas the resident microorganisms contribute more than 1 million genes (11). The human microbiome represents the entire population of microorganisms, including bacteria, viruses, fungi, archaea, and small protozoa, along with their material and metabolic products, that mainly inhabit the epithelial barrier surfaces of the human body, such as the skin, lung, gastrointestinal tracts, mucous membranes of the oral cavity, and vagina (12). Accordingly, these microorganisms have a long history of co-evolution with humans and are inextricably linked with human physiology.

The intestinal bacteria are of three types: beneficial, harmful, and neutral. The intestinal or gut microbiome is highly diverse and plays a role in synthesizing many essential vitamins and amino acids while promoting the digestion and absorption of various minerals. Moreover, the gut microbiome plays an important role in maintaining an autoimmune barrier and enhances the mucosal immune system. Thus, intestinal dysbiosis can affect the nervous and immune systems by interacting with the Vagus nerve and by destroying the integrity of the epithelium, respectively (13). Abnormal variations in the intestinal microbiome is a key factor in the development of many metabolic diseases such as obesity, type-2 diabetes, and atherosclerosis (14). In addition, gut microbes play an important role in constipation, diarrhea, colitis, chronic gastritis, urinary tract infections, skin aging, acne, osteoporosis (15), allergic diseases (16), liver cirrhosis (17), depressive disorder (18), cardiovascular diseases (19), lung diseases (9), autoimmune diseases (20), central nervous system disorders (21), and cancers (22). Therefore, the effect of the intestinal microbiome on the body's immune system is not just limited to the intestinal tract but is spread across the entire body. This effect mainly manifests in two aspects: [1] promoting the development of the host immune system and [2] regulating the immune system. For example, dendritic cells (DCs) recognize intestinal bacteria and activate appropriate signaling pathways to produce different cytokines through Toll-like receptors (TLRs), which then regulate the differentiation of T cells into different subgroups to achieve the balance between bacterial tolerance and immunity. The metabolic products from microorganisms such as short-chain fatty acids, bile acids, vitamins, polyamines, lipids, and histamines can also affect the immune responses (23–25).

## Human Microbiome and Cancers

**Figure 1:**
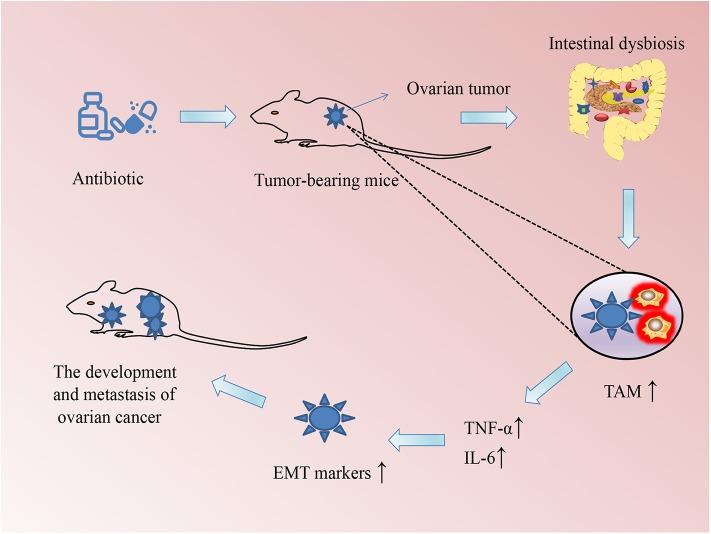
Schematic of the association between changes in the microbiome and the development of ovarian cancer in mice. Intestinal dysbiosis (ID) was induced by drinking water supplemented with a mixture of antibiotics every day so that their intestinal flora was completely exhausted. The ID could promote macrophages to polarize to tumor-associated macrophages (TAMs), which secrete the inflammatory cytokines tumor necrosis factor (TNF)-α and interleukin (IL)-6. These cytokines could accelerate the growth of xenograft ovarian tumors in mice through promoting epithelial–mesenchymal transition (EMT).

Increasing evidence is pointing to the role of microbiome in the occurrence and development of a variety of cancers. For example, intestinal microbiome imbalance is related to the occurrence of gastrointestinal tumors such as esophageal cancer, gastric cancer, colorectal cancer, and gallbladder cancer (26). Moreover, tumors in other parts of the body, including hepatocellular carcinoma, breast cancer, pancreatic cancer, prostate cancer, and ovarian cancer, occur and grow because of disturbances in intestinal microbiome (10, 27, 28). Gut dysbiosis may be a potential link among cancer risk, aging, and inflammation (29). For example, infections caused by *Helicobacter pylori*, fusobacteria, and other potentially infectious pathogens may also increase the risk of colorectal cancer (30, 31). *Helicobacter pylori* infections increase the risk of developing gastric cancer and pancreatic cancer (32); however, they reduce the risk of esophageal adenocarcinoma (33). Some specific bacteria such as proteobacteria, firmicutes, and bacillus found in the breast tissue, skin, intestinal tract, and oropharynx have been proposed to contribute to breast carcinogenesis (27). Researchers from the Mayo Clinic Department of Breast Surgery found bacterial DNA in breast cancer tissue under sterile conditions and showed that the microbiome in women with breast cancer is different from that of women without breast cancer (34). A population-based nested case-control study evaluated the relationship between the oral microbiome and the risk of pancreatic cancer and demonstrated that the presence of *Porphyromonas gingivalis* and *Aggregatibacter actinomycetemcomitans* is positively correlated with pancreatic cancer, whereas the presence of bacteria belonging to the phylum Fusobacteria and its genus *Leptotrichia* is related to a reduced risk of pancreatic cancer (35). Another study on tumor microbiome diversity of pancreatic adenocarcinoma showed that gut microbiome influences not just the host immune response but also the natural history of pancreatic cancer (36). Intestinal dysbiosis stimulates the growth of xenograft tumors and induces the development of epithelial-mesenchymal transition in ovarian cancer cells, which activates TAM via the secretion of IL-6 and TNF-α (37) (Figure 1). These microbes not only produce toxic metabolites or carcinogenic compounds that directly contribute to the occurrence of cancer but can also lead to the occurrence of cancer via promoting inflammation or immune suppression. Conversely, some cancers may disturb and change the microbiome composition in the human body during disease development and treatment. In a clinical trial, autologous transplantation of fecal microbes was performed in patients with acute myeloid leukemia undergoing chemotherapy and antibiotic intake to restore the balance of the gut microbiome and eliminate resistant bacteria induced by the treatment (NCT02928523). Several other clinical trials of microbiome-based studies have been completed or are ongoing to elucidate the important role of microbiome in cancers (see Table 1).

**Table 1:** Clinical trials of microbiome-based studies in cancer.

**Study title**	**Disease**	**Intervention**	**Main findings**	**NCT**
The oral microbiome and upper aerodigestive squamous cell cancer	Oral cancer	Classification of the oral Microbiome using a 16S rRNA gene sequence-based approach and taxonomic approach	Species from 11 phyla have been identified: Firmicutes, Bacteroidetes, Proteobacteria, Actinobacteria, Spirochetes, Fusobacteria, TM7, Synergistetes, Chlamydiae, Chloroflexi, and SR1	NCT03304132
Oral microbiome and pancreatic cancer	Pancreatic cancer	16S rRNA gene sequencing assay	A history of periodontal disease and the presence of circulating antibodies to selected oral pathogens have been associated with an increased risk of pancreatic cancer; however, direct relationships of oral microbes with pancreatic cancer have not been evaluated in this prospective study.	NCT03302637
Microbiome test for the detection of colorectal polyps and cancer	Colorectal neoplasms colorectal cancer	No description	The limited sample size is suggested to be insufficient to quantify the sensitivity to colorectal cancer.	NCT02141945
Colon cancer study of fecal samples in Shanghai, China	Colorectal cancer	No description	The main difference was a 3-fold higher median relative abundance of Proteobacteria. Antibiotic exposure and other potential confounders did not affect the associations.	NCT01778595
Study of gut microbiome and colorectal tumors	Serrated polyp hyperplastic polyp sessile serrated adenoma colorectal cancer	Diagnostic Test: Mobio PowerSoil DNA Isolation Kit	Prevalence and relative abundance of selected fecal microbial taxa	NCT03297996
An evaluation of probiotic in the clinical course of patients with colorectal cancer	Colorectal cancer	Dietary Supplement: Probiotic Other: Placebo	Level of circulating inflammatory cytokines (TNF-α, IFN-γ, IL-6, IL-10, IL-12, IL-17A, IL17C &IL-22) pre and post intervention. [Time Frame: change from pre intervention baseline level at post 6 months intervention]. The level of eight colorectal cancer-related inflammatory cytokines (pg/mL) were measured and compared pre and post intervention in both the probiotic and placebo groups.	NCT03782428
Intestinal bacteria and breast cancer risk	Breast neoplasms	No description	Postmenopausal women with breast cancer have an altered composition and estrogen-independent low diversity of their gut microbiota. However, the influence on breast cancer risk and prognosis is unknown.	NCT01461070
Role of microbiome as a biomarker in locoregionally advanced oropharyngeal squamous cell carcinoma	Locoregionally advanced oropharyngeal squamous cell carcinoma	No description	This study does not involve any therapeutic intervention. The study includes a prospective cohort of up to 60 patients diagnosed with locoregionally advanced oropharyngeal squamous cell carcinoma (LA-OPSCC) treated with chemoradiotherapy (CRT) at Princess Margaret Cancer Center.	NCT03759730
*Escherichia coli* Nissle in oncology	Gastric cancer colorectal cancer	Drug: *E. coli* Nissle suspension Drug: Placebo	Treatment with 5-fluoruracil in combination with other chemotherapeutic remedies (FLO, FOLFOX, FOLFOX-Bev, FOLFIRI) is planned, and the effect of an *E. coli* Nissle suspension on the duration and intensity of chemotherapy induced diarrhea will be evaluated.	NCT02706184
Prevention of dysbiosis complications with autologous fecal microbiota transplantation in acute myeloid leukemia patients undergoing intensive treatment	Leukemia, myeloid, acute	Autologous fecal microbiota transplantation	Evaluation of AFMT efficacy in dysbiosis correction based on the microbiota diversity and evaluation of AFMT efficacy in MDRB eradication based on bacterial culture	NCT02928523

## Microbiome and Estrogen in Ovarian Cancer

Abnormal estrogen synthesis and metabolism may be related to the occurrence and development of ovarian cancer. One prospective study showed that women with the highest levels of circulating estradiol had three times higher risk of developing ovarian cancer (odds ratio = 3.0, 95% confidence interval: 0.6–14.9) than women with the lowest estradiol levels (38). In addition, this study showed that after using menopausal hormone therapy for five or more years, the risk of developing ovarian cancer becomes high for at least five years, even after ceasing the treatment. Conversely, the use of progestins was suggested to reduce the formation of ovarian epithelial lesions caused by estrogen (39).

One study investigated the relationship of the diversity and composition of the fecal microbiome with urinary estrogens and estrogen metabolites and found that postmenopausal women with a more diverse gut microbiome had a higher proportion of hydroxylated estrogen metabolites in their urine (40). This finding implied that the gut microbiome may influence the level of estrogen produced. The influence of the gut microbiome on the formation and development of ovarian cancer via affecting the level of estrogen remains highly controversial, although two main mechanisms have been proposed to explain this potential relationship: [1] the gut microbiome disturbs the enterohepatic circulation of estrogen (41), or [2] interferes with the secretion of β-glucuronidase, an enzyme that degrades the active forms of estrogen (42). Through these mechanisms, the microbiome could increase the level of estrogen, which would, in turn, promote gene transcription and mitotic activities. In addition, high activity of β-glucuronidase may induce the production of carcinogens, which can lead to oncogenesis (43).

## Microbiome, Host Recognition Receptors, and Ovarian Cancer

Microorganisms mainly interact with human cells through the combination of microbe-associated molecular patterns on the microbial surface and corresponding receptors of the host (44). These important recognition receptors on the host cell include pattern recognition receptors (PRRs), TLRs, nucleotide-binding oligomerization domain-containing protein-like receptors (NLRs), and C-type lectin receptors (CLRs) (45).

Nuclear factor-kappa B (NF-κB) is the downstream target of TLR, which can stimulate the release of inflammatory cytokines. A hallmark of innate immunity is the proper recognition of potential pathogens and harmless antigens through PRRs. Activation of PRRs has been suggested as a cause of chronic inflammation and carcinogenesis (46). For example, the upregulation of PRRs in endometriosis can lead to progression into ovarian endometrioid carcinoma and clear cell carcinoma (47). In particular, carcinogenesis can be accelerated by PRR-induced activation of hepatocyte nuclear factor 1 signaling and dependent oxidative stress (48). TLRs belong to the PRR system and can recognize highly conserved structural motifs of pathogenic microorganisms (49). The relationship between TLR and cancers has thus far been studied in prostate cancer (50), breast cancer, hepatocellular carcinoma (51), pancreatic carcinoma, esophageal squamous cell carcinoma (52), and nasopharyngeal cancer (53). TLRs have also been suggested to play a role in the response to ovarian cancer treatment. Indeed, chemotherapy resistance is the primary factor contributing to the long-term treatment and affecting overall survival of ovarian cancer patients, and not gene mutations (54). TLRs are expressed on the surface of ovarian cancer cells, and their activation and consequent signaling cascades can lead to paclitaxel chemoresistance. It can also contribute to the growth of ovarian tumors (55). Ovarian granulosa cell tumors are the most common sex-chord neoplasm among ovarian cancers, in which NF-κB can be activated by TLR4 (56).

NLRs are the innate immune PRRs that can activate multiple pro-inflammatory responses and lead to antigen-specific acquired immunity via downstream signaling. NLRs are involved in the occurrence, development, and pathology of cancers such as glioblastoma, prostate adenocarcinoma, breast cancer, melanoma, and colon adenocarcinoma (57). NLRP3 is a pyrin domain-containing protein of the NLR family and plays a vital role in the proliferation and migration of lung cancer cells (58). Macrophages and natural killer (NK) cells are important immune cells in the occurrence and development of cancers, including ovarian cancer. Nucleotide-binding oligomerization domain-containing protein 1 [(NOD1) and 2 (NOD2)] belong to the NLR family. NOD1 and NOD2 can induce an increase in IL-6 and NF-κB, which can enhance the metastasis and drug resistance of ovarian cancer (59, 60).

CLRs, which harbor at least one C-type lectin-like domain, play an important role in antifungal and antibacterial immune responses, regulation of immune homeostasis, autoimmunity, allergy, and recognition of dead and cancerous cells (61). CLRs participate in immune escape in conjunction with tumor-associated macrophages TAMs presented in ovarian cancer tissue (62).

## The Pelvic Microbiome and Ovarian Cancer

In addition to treatment resistance, late-stage diagnosis is a primary reason behind high mortality rate of patients with ovarian cancer. The disease is often diagnosed late mainly because of the lack of specific symptoms. One of the main hallmarks of cancer is the chronic inflammatory state. High-grade serous carcinoma (HGSC) with a high mortality rate is the most common subtype of ovarian cancer. Preventative salpingectomy in high-risk populations could prevent HGSC as well as endometrioid and clear cell carcinoma. It inhibits the spread of potentially cancerous cells into the ovarian/peritoneal surfaces and the ascension of endometrium and related inflammatory factors (63). However, preventive salpingectomy is not the first treatment in the high-risk population. Rasmussen et al. (64) found that a history of PID was positively correlated with the development of ovarian borderline tumors but not invasive ovarian cancer. Subsequently, they also showed that PID slightly increased the risk of serous ovarian cancer (65). Another study based on 16S rRNA high-throughput sequencing reported that the ratio of Proteobacteria/Firmicutes was significantly increased in ovarian cancer tissues (5). This result showed that changes in the microbiome might be associated with the development of ovarian cancer. This study may offer a way to prevent ovarian cancer. For example, the use of Proteobacteria vaccine may reduce the incidence of ovarian cancer.

*Chlamydia trachomatis* and *Mycoplasma genitalium* are sexually transmitted microorganisms associated with pelvic inflammatory disease. The presence of chlamydial HSP60-1 IgG antibodies is associated with high-grade ovarian epithelial carcinoma, while the presence of *M. genitalium* IgG antibodies is associated with borderline ovarian tumors (66). One case-control study including 50 patients with ovarian cancer and 30 control subjects with non-malignant ovarian lesions showed that human papillomavirus (HPV) 16 infection was significantly higher in cancer tissues than in the non-malignant tissues of the control group (67). Similarly, the high-risk HPV types 16, 18, and 45 were found to be associated with ovarian cancer in Saudi patients (68). However, Ingerslev et al. (69) concluded that high-risk HPV was not associated with epithelial ovarian cancer in a Caucasian population. Therefore, the association between HPV infection and ovarian cancer might differ according to ethnicity or genetic background, and thus more studies are warranted to clarify these potential links. For epithelial ovarian carcinoma, the chances of infection with *C*. *trachomatis* and cytomegalovirus were 32 times and 8 times greater than normal ovaries tissues, respectively (70). These examples demonstrating the relationship between the microbiome and ovarian cancer are summarized in Table 2. A clinical trial comparing the pelvic microbiome between benign and malignant ovarian diseases is now recruiting, which plans to conduct 16S rRNA sequence analysis and microculture of discharges/flushing fluids from the vagina, feces, and fimbrial end of the fallopian tube (NCT03388996).

**Table 2:** Examples of association between microbiome and ovarian cancer.

**Cancer type**	**Microbiome changes**	**References**
Ovarian cancer	The ratio of the two phyla Proteobacteria/Firmicutes was notably increased in ovarian cancer.	Zhou et al. (5)
Ovarian cancer	Intestinal dysbiosis induced by high dose antibiotics promotes epithelial–mesenchymal transition by activating tumor-associated macrophages in ovarian cancer.	Xu et al. (37)
Ovarian borderline tumor	The history of PID is associated with an increased risk of ovarian borderline tumors	Rasmussen et al. (64)
Serous ovarian cancer	Pelvic inflammatory disease was associated with a modestly increased risk of serous ovarian cancer, but not of other histotypes.	Rasmussen et al. (65)
Ovarian cancer	Chlamydial HSP60-1 lgG antibodies chlamydial HSP60-1 IgG antibodies were associated with type II ovarian cancer	Idahl et al. (66)
Ovarian borderline tumor	*Mycoplasma genitalium* lgG antibodies were associated with borderline ovarian tumors	Idahl et al. (66)
Ovarian cancer	HPV 16 infection was significantly higher in cancer tissues compared to controls	Wu et al. (67)
Ovarian cancer (in Saudi patients)	The high-risk HPV types 16, 18, and 45 were highly associated with the advanced stages.	Al-Shabanah et al. (68)
Ovarian cancer	*Chlamydia trachomatis*-positive was 32 times greater for cases with epithelial ovarian carcinoma than for those with normal ovaries. Cytomegalovirus positive were 8 times greater for cases with epithelial ovarian carcinoma than for those with normal ovaries.	Shanmughapriya et al. (70)
Ovarian cancer	The presence of ovarian cancer or a germline BRCA1 mutation favored a composition of the cervicovaginal microbiota (i.e., a type O community) that is more commonly seen in older than in younger women.	Nene et al. (71)

*PID, pelvic inflammatory disease; A type O community: lactobacilli accounted for <50% of the species present in the cervicovaginal microbiota*.

## The Microbiome as a Biomarker for Cancers

It is important to identify some non-invasive biomarkers for predicting the risk factors of cancer and monitoring the treatment progress. As mentioned above, the changes in microbiome may lead to the occurrence and development of cancer, whereas some cancers may disturb and change the microbiome composition. The microorganisms and their metabolites exist in various body fluids such as saliva, blood, urine, feces, and cervicovaginal discharge/flushing fluid. Therefore, the microorganisms or their metabolites can be novel and non-invasive biomarkers for cancers. Farrell et al. found that 31 bacterial species/clusters are increased and 25 bacterial species/clusters are decreased in the saliva of patients with pancreatic cancer in comparison to healthy controls (72). Liu et al. showed that patient groups with lung cancer have elimination, low-density, and loss of bacterial diversity microbial ecosystem in gut, which indicates that gut microbiome may serve as a biomarker in lung cancer (73). Sobhani et al. found that colorectal cancer-associated microorganisms, which can increase the number of hypermethylated genes in murine colonic mucosa compared with the healthy group, may become the potential biomarkers for colorectal cancer (74). Another study about non-small cell lung cancer showed that hippuric acid, a metabolite from the microbiome, is higher in the PD-1 blockade therapy responders than in non-responders (75). Thus, hippuric acid can serve as a combinatorial biomarker to screen the patients who are more suitable for cancer immunotherapy and the non-responders are advised to choose other more effective therapies. In addition, colitis is an adverse event associated with the use of PD-1 inhibitors in patients with cancer. Thus, a clinical trial (NCT02600143) was conducted to identify a useful predictive biomarker in the microbiome that can distinguish patients who are more prone to colitis. Distinct genetic profiles based on 99 loci that are different between the patients who developed immunotherapy-induced colitis and those who did not were identified through this trial. Patients with ovarian cancer or a germline BRCA1 mutation are more prone to have a different proportion of lactobacilli in cervicovaginal microbiome (<50% of species present in the healthy controls). Further, this observation is more commonly seen in older women than in younger women (71) (also shown in Table 2). Further research is needed on the use of microbiome as biomarkers in ovarian cancer.

## The Microbiome in Cancer Treatment

**Figure 2:**
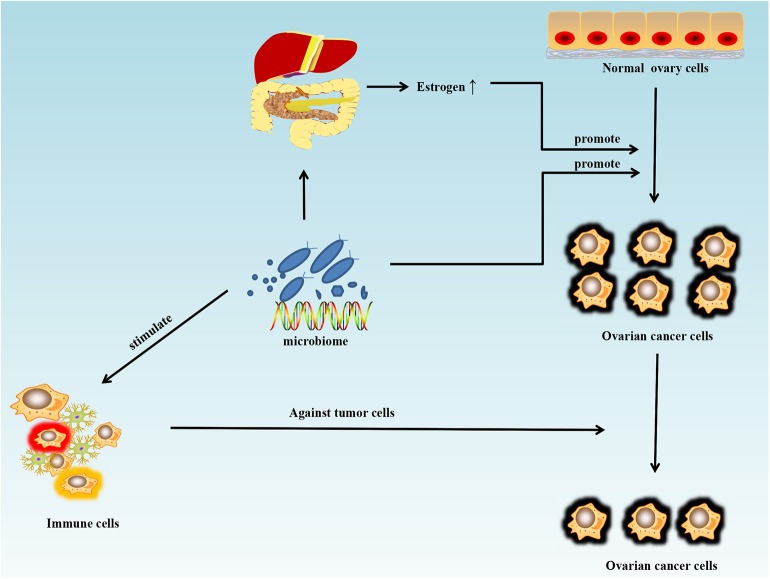
Schematic of the influence of the microbiome on ovarian cancer. Antibiotic use can disturb the normal states of some microorganisms in the human body and may increase the risk and promote the growth of ovarian cancer. Estrogen can promote the growth of ovarian cancer by disturbing the metabolism of estrogen in the liver and intestines. However, the microbiome can also stimulate the immune system and increase anti-tumor activity.

With the development of metagenomics, transcriptomics, proteomics, metabolomics, and particularly, 16S rRNA sequencing, the importance of microorganisms has become increasingly recognized and their roles in maintaining health and triggering various diseases are being identified. Microorganisms function like a double-edged sword in tumor chemotherapy and targeted therapy. On one hand, microorganisms and their metabolites may stimulate the immune system and increase the anti-tumor activity. On the other, certain metabolites of microorganisms can weaken the therapeutic effect of drugs. Cyclophosphamide is an anti-cancer drug that can be used in ovarian cancer, and its therapeutic efficacy partly depends on the ability to stimulate anti-tumor immune responses. One study showed that the gut microbiome can enhance the anti-tumor immune response through stimulating the generation of “pathogenic” T helper 17 cells and memory Th1 immune responses (76). Sivan et al. (77) compared the growth of melanoma in mice harboring distinct commensal microbiota and observed differences in spontaneous antitumor immunity effects to anti-PDL-1, which were eliminated upon cohousing or after fecal transfer. Compared with the non-Bifidobacterium-treated group, the tumor control of mice treated with Bifidobacterium was significantly improved. In this mouse model, tumor-specific T cells around the tumor were strongly induced and the accumulation of tumor-specific CD8^+^ T cells was also increased. T cell responses specific against Bacteroides species (*B. thetaiotaomicron* or *B. fragilis*) in mice and patients could also favor the efficacy of CTLA-4 blockade (78). The microbiome could offer a new treatment strategy for ovarian cancer by stimulating the immune system and increasing anti-tumor activity (Figure 2). Microorganisms are also known to interfere with therapeutic agents. For example, some bacteria can metabolize the chemotherapeutic drug gemcitabine and thus lower its therapeutic efficacy (79).

Chemotherapy resistance is an important aspect of cancer treatment difficulty. Microbiome may contribute to the development of such resistance (80). Yu et al. found that *Fusobacterium nucleatum* is not only related to colorectal cancer recurrence but also promotes chemoresistance by inducing autophagy (81). Gammaproteobacteria is a group of bacteria that commonly exist in pancreatic cancer tissues. Bacteria of this group can reduce the response to gemcitabine by expressing the cytidine deaminase enzyme (78). Butyrate from gut microbiome can decrease the expression of histone deacetylases (HDACs) (82). The elevated expression of HDAC2 can reduce the sensitivity of doxorubicin in colorectal cancer (83), cisplatin in non-small cell lung cancer (84), and temozolomide in glioblastoma cells (85). Cisplatin and paclitaxel can be usually used in ovarian cancer chemotherapy. Asfaha et al. reported that the novel HDAC inhibitors can increase the sensitivity of cisplatin in cisplatin-resistant cancer cells (86). Another study showed that *P. gingivalis* can induce paclitaxel resistance through activating the Notch1 pathway in oral squamous cell carcinoma (87).

Owing to the important role of PRRs in carcinogenesis, targeting PRRs may become an effective cancer-preventive strategy. TLR signaling can promote the maturation of DCs, enhance the ability of antigen presentation, and amplify the functions of CD8^+^ T cytotoxic effectors. Inhibiting TLR signaling may be an immunotherapy strategy in cancer treatment (88). The immune checkpoint-targeted anti-CTLA-4, anti-PD-1, and anti-PD-L1 monoclonal antibodies have shown clinical benefits for patients with some types of cancer; however, these benefits are not seen in all patients and all types of cancers. Nonetheless, the primary and secondary resistance acquired during administration of these antibodies can be overcome by combining them with the PRR agonists (89).

The main treatments for ovarian cancer are cytoreductive surgery and chemotherapy such as platinum and taxane chemotherapy. Some targeted therapies, including anti-angiogenic drugs, poly-ADP ribopolymerase inhibitors (PARPi), and immune checkpoint inhibitors, have shown good performance in delaying recurrence and prolonging the overall survival time of patients. For example, ovarian cancer patients with BRCA1/2 mutations treated with PARPi therapy showed an ~4-fold longer progression-free survival compared with those taking the placebo, whereas the increase for patients without the BRCA1/2 mutation was ~2-fold (90). However, the use of PARPi may not avoid developing drug resistance, and more effective targeted therapies are required to prevent drug resistance. In addition, patients with ovarian cancer having germline BRCA1 mutation are more likely to have reduced proportion of lactobacilli (<50% of the total microbial species) in cervicovaginal microbiome (71). Further studies about the relationship among PARPi, BRCA1, and the abundance of lactobacilli in the cervicovaginal microbiome are warranted.

Cisplatin is used as a first-line treatment for advance-stage ovarian cancer. Intestinal damage is an adverse effect associated with cisplatin, which results in modifications in the process of treatment, such as dose reduction and delaying or cessation of the treatment (91). Alfredo Perales-Puchalt et al. found that reconstitution of the intestinal microbiome in cisplatin-treated mice accelerates intestinal healing (92).

In short, microbiome can change anti-tumor activity, mediate chemotherapy resistance, and reduce the number and intensity of adverse effects in the process of cancer treatment.

## Conclusion, Challenges, and Perspective

The microbiome plays a crucial role in maintaining the health of the human body, and abnormalities in the microbiome have been associated with a variety of diseases, including ovarian cancer. With the development of technologies such as high-throughput DNA and 16S rRNA sequencing, more and more aspects of the human microbiome are being revealed. Probiotic bacteria show the potential to become a significant component in the prevention and treatment of cancers (76). Despite increasing studies pointing to a relationship between the microbiome and ovarian cancer, the mechanisms underlying this relationship have not yet been fully elucidated. Particularly, the specific microorganisms involved in the pathogenesis of ovarian cancer are not identified. Further studies are warranted to examine the clinical feasibility and value of using the microbiome as a biomarker for diagnosis. The microbiome also shows good potential as a treatment strategy, which can help to overcome the current lack of an effective treatment for advanced, recurrent, and chemo-resistant ovarian cancer. Treatments that activate the innate immune system have shown promising results; such treatments effectively suppress or destroy the cancer cells by activating the immune system response. Given the important immune function of the microbiome, the focus of microbiome-based therapy has shifted from regulating the microbiome to developing microbial monocultures that can regulate the immune system. Future research efforts in this regard should focus on identifying specific metabolites and immunomodulatory molecules produced by the microorganisms and the specific components of the microbiome linked to different subsets of ovarian cancer.

## Author Contributions

HC drafted the manuscript. HC and ZW were responsible for the conception, planning and carrying out the study, reviewing the literature. YW, LC, XC, and FG were responsible for discussing and final review of this manuscript. HY was responsible for the conception and final review of this manuscript. All authors read and approved the final manuscript.

### Conflict of Interest

The authors declare that the research was conducted in the absence of any commercial or financial relationships that could be construed as a potential conflict of interest.

## Abbreviations

OvCa: ovarian cancer
PID: pelvic inflammatory disease
HP: helicobacter pylori
AFMT: autologous transplantation of fecal microbes
AML: acute myeloid leukemia
MHT: menopausal hormone therapy
MAMPs: microbe-associated molecular patterns
PRRs: pattern recognition receptors
TLRs: Toll-like receptors
DCs: dendritic cells
NOD: nucleotide-binding oligomerization domain
NLRs: NOD-like receptors
IDB: intestinal dysbiosis
TAM: tumor-associated macrophage
TNF-α: tumor necrosis factor -α
IL-6: interleukin−6
EMT: epithelial-mesenchymal transition
CLRs: C-type lectin receptors
NK: natural killer
CTLD: C-type lectin-like domain
HDAC: histone deacetylases
HGSC: high-grade serous carcinoma
EOC: epithelial ovarian cancer
CMV: cytomegalovirus
PARPi: poly ADP ribopolymerase inhibitors.
